# Investigation of a HAP-UAV Collaboration Scheme for Throughput Maximization via Joint User Association and 3D UAV Placement

**DOI:** 10.3390/s23136095

**Published:** 2023-07-02

**Authors:** Huda Goehar, Ahmed S. Khwaja, Ali A. Alnoman, Alagan Anpalagan, Muhammad Jaseemuddin

**Affiliations:** 1 Department of Electrical, Computer and Biomedical Engineering, Toronto Metropolitan University, Toronto, ON M5B 2K3, Canada; hgoehar@torontomu.ca (H.G.); kashaharyar@gmail.com (A.S.K.); jaseem@torontomu.ca (M.J.); 2 Department of Computer Science and Engineering, American University of Ras Al Khaimah, Ras al Khaimah P.O. Box 10021, United Arab Emirates; ali.alnoman@aurak.ac.ae

**Keywords:** high-altitude platform (HAP), unmanned aerial vehicles (UAVs), HAP-UAV collaboration, 3D placement, user association

## Abstract

In this paper, a collaboration scheme between a high-altitude platform (HAP) and several unmanned aerial vehicles (UAVs) for wireless communication networks is investigated. The main objective of this study is to maximize the total downlink throughput of the ground users by optimizing the UAVs’ three-dimensional (3D) placements and user associations. An optimization problem is formulated and a separate genetic-algorithm-based approach is proposed to solve the problem. The K-means algorithm is also utilized to find the initial UAV placement to reduce the convergence time of the proposed genetic-algorithm-based allocation. The performance of the proposed algorithm is analyzed in terms of convergence time, complexity, and fairness. Finally, the simulation results show that the proposed HAP-UAV integrated network achieves a higher total throughput through joint user association and UAV placement schemes compared to a scheme with a single HAP serving all users.

## 1. Introduction

Due to the high capabilities of unmanned aerial vehicles (UAVs) in terms of mobility, dynamic wireless coverage, and ability to reach remote areas, UAVs have been utilized in a variety of applications especially those characterized by hard conditions and relatively difficult to reach by humans, such as during rescue missions [1]. The UAVs have been widely employed in various applications, including mapping, fire detection, gaming, traffic monitoring, surveillance, etc. [2]. In recent years, UAVs have been extensively integrated into wireless communication networks due to their mobility, low cost, on-demand deployment, and flexibility [3]. The UAVs can also perform as aerial base stations (BSs) to support the existing terrestrial wireless networks. The main advantage of using UAVs as aerial BSs is connectivity, coverage, and reliability improvement of wireless networks. The UAVs can change their positions, avoid obstacles, and increase the possibility of establishing line-of-sight links (LoS) with users, which allows them to provide better service when serving as flying BSs.

In addition, UAVs can function as relays to improve connectivity and capacity and save infrastructure expenditure in rural or disaster areas [4]. However, when there is any fault in the terrestrial network, UAVs cannot provide reliable and continuous connectivity to ground users due to their power and coverage limitations [5]. Therefore, designing a framework that involves both high-altitude platforms (HAPs) and UAVs has emerged to guarantee reliable connectivity to ground users with better data rate performance compared to terrestrial networks. Moreover, the utilization of HAPs as BSs can also provide better connectivity compared to terrestrial BSs. This is due to the fact that HAPs are placed at a high altitude, which enables them to provide enormous coverage and establish LoS channels with UAVs and terrestrial users. The HAPs can also be equipped with solar panels to generate their power requirements. Compared to low earth orbit (LEO) satellites, HAPs require less maintenance and lower deployment expenses.

Thus, HAP-UAV integrated networks can have promising achievements in future wireless communication. Such integrated networks are considered cost-effective and fast-deployment solutions for providing connectivity to areas that require additional capacity or temporary coverage, such as concerts, festivals, and sports events. The HAPs can be considered centralized controllers that manage and configure the entire network, while the UAVs can function as relays to increase coverage and users’ data rate. Nevertheless, there are several challenges facing the HAP-UAV integrated network implementation, including 3D UAV placement optimization, energy constraints, flight duration, interference management, user association, and bandwidth allocation.

### 1.1. Related Work

The optimal deployment of unmanned aerial vehicles is a crucial design consideration since the UAVs can move in three dimensions (3D). The 3D placement of UAVs is challenging because it depends on many factors, including the air-to-ground (A2G) channel model, deployment environment, and ground users’ locations [6]. Moreover, when it comes to a multiple-UAV wireless network, UAV placement becomes more challenging due to the inter-cell interference which affects the system performance. In addition, the placement of UAVs affects the transmit power, coverage, and QoS significantly, which makes 3D placement an essential parameter in resource management. Furthermore, according to their objectives, UAVs can be deployed in different modes, such as 3D, 2D, single-UAV, or multi-UAV. The objectives could be maximization of quality of service (QoS), sum-rate, power, signal-to-noise ratio (SNR), throughput, minimization of the number of UAVs, etc.

The paper [7] addressed the UAV placement problem and proposed a plan to minimize the number of UAVs while maximizing the coverage area. The authors proposed a heuristic algorithm based on particle swarm optimization (PSO) considering different densities of areas. It was observed that the UAVs can increase their coverage area by increasing their altitude in the high-density areas, while decreasing their altitude helps to reduce interference. Similarly, the authors in [8] proposed a PSO-based algorithm to find the optimal UAV positions while minimizing the number of UAVs.The authors in [9] proposed a UAV placement scheme considering the backhaul peak rate and bandwidth of UAVs by introducing two approaches, namely, network-centric and user-centric. The network-centric approach is aimed to maximize the number of users, whereas the user-centric approach aims to maximize the users’ sum-rate. Furthermore, the authors in [10] proposed an artificial-neural-networks-based solution to UAV placement for enhancing an integrated UAV-D2D Non-orthogonal Multiple Access (NOMA) cooperative network. There are still many other challenges associated with UAV placement, such as weather conditions, battery lifetime, path loss, height, and interference.

The UAV placement and user association problems have been addressed in several papers in the literature to realize efficient UAV-assisted networks. The study in [11] jointly optimized the 3D positioning of UAVs and bandwidth allocation in order to minimize the cumulative power using an iterative algorithm. In the same context, the authors in [12] proposed a 3D UAV base station placement to maximize the number of covered users while minimizing the transmission power. The authors in [13] used full duplex communication in UAVs aiming to maximize the uplink and downlink sum rate. A distributed reinforcement learning approach was applied in [14] to achieve optimal dynamic user association while minimizing the users’ transmit power in UAV-assisted air-ground networks. In [15], the authors investigated the effect of the UAVs’ altitude and the number of UAVs on the cumulative rate maximization in UAV-assisted wireless networks utilizing stochastic geometry. The study in [16] addressed the overall system throughput optimization by determining the optimal UAV positioning. Here, the authors presented two methods, namely, a heuristic method and an approximation algorithm considering the users’ positions and the data demands.

Furthermore, in [17], the authors aimed to optimize user association and UAV placement while maximizing the total downlink throughput for users in a UAV-enabled network, considering LoS and non-LoS (NLoS) links between UAVs and users. The authors proposed three approaches and compared their performance. The first approach applied the binary log-linear learning algorithm (BLLL), which is a game theoretic learning algorithm. The BLLL algorithm provided a global-optimal solution but had an exponential convergence since each UAV needed a complete knowledge of all users’ throughput. Thus, a considerable amount of information was needed to be exchanged in the network. In the second approach, the optimization problem was reformulated as a submodular optimization problem and solved using the greedy algorithm that guaranteed performance. The third approach applied a heuristic greedy algorithm that required less information and reached the optimal solution in a few iterations. There was no performance guarantee yet promising results could be obtained. The study in [18] addressed the user association, 3D UAV placement, and bandwidth allocation optimization problem to maximize the data rate in UAV-enabled networks considering wireless backhaul communication between the UAV BSs and a macro-BS. The authors in that study employed a decomposition method to optimize user association and wireless backhaul bandwidth allocation. Subsequently, a heuristic PSO algorithm was used to find the optimal 3D UAV placement.

All the aforementioned papers considered UAV-assisted networks in the absence of a HAP. Few research works in the literature have addressed the data rate maximization problem for networks in the presence of both HAP and UAVs. The authors in [19] considered a scenario consisting of a HAP, multiple UAVs, and on-ground fog computing nodes. The HAP was used to charge the UAVs via laser beam transmission from the former to the latter. The authors proposed a strategy to maximize the overall utility of the UAVs by offloading part of their tasks and energy to the fog computing nodes. In a similar context, the authors in [1] considered using a HAP to accommodate the computing tasks offloaded from UAVs. The work aimed to minimize the computing latency by optimizing the offloading decision, transmit power, and uplink signal splitting ratio. Likewise, the authors in [20] proposed a vehicular computing network in which on-ground vehicular nodes could offload their tasks to the HAP to minimize the computing delay by optimizing the offloading rate, signal split rate, and transmit power. In order to improve the service satisfaction and energy consumption in joint Internet of Things (IoT) and HAP-UAV networks, the authors in [21] considered optimizing device association, partial offloading, and communication resources. In [22], the authors proposed a HAP-UAV integrated network using the NOMA technology. The work employed a power management scheme to minimize the transmission distortion between the HAP and UAVs to the users. Placing reconfigurable intelligent surfaces (RIS) on HAPs was proposed in [23] to provide service to users that could not be served by terrestrial networks. The work employed an approach to maximize the number of users, while minimizing the total power consumed by the involved control station and RIS.

In [24], the authors proposed a system model with ground users on vehicles, UAVs, and HAPs. The work considered calculating the optimal ratio to offload tasks by the ground users to the UAVs and HAPs such that the total processing time was minimized. In [5], the authors proposed a double-layer cooperated scheme with one HAP and multiple UAVs. The authors divided the users into clusters such that each cluster was served by a UAV that received data from the users and forwarded it to the HAP. The study aimed to jointly optimize channel allocation, users’ power, and UAVs’ altitudes while achieving a high data rate by applying the K-means algorithm [25], successive convex approximation, and the ant colony optimization approaches. The study, however, did not consider any direct association between the users and the HAP. In [26], the authors proposed a scenario involving the integration of UAV-HAP-BS for maximizing the downlink sum rate. The authors considered the deployment of HAP and UAV and sub-channel allocation. Nevertheless, the utilized system model did not consider direct links between the HAP and users on the ground. In [27], the authors proposed a deep reinforcement learning approach for 3D UAV placement in a HAP-UAV integrated network to achieve fairness in data rates among users and to maintain load balancing among the UAVs. Here, the users could be connected to either the HAPS or the UAVS, and both the HAPS and UAVS were provided backhaul connectivity through a LEO satellite. However, the work did not consider any connection between the HAP and UAV.

### 1.2. Contributions

In this paper, we propose a double-layer cooperative scheme in which multiple UAVs and one HAP collaborate to serve users in a given area. Our work considers using UAVs as relays to enhance the total throughput of the system and provide users with a better service. We consider that the users can be served by either the UAVs or directly by the HAP. The main objective of this study is to maximize the total downlink throughput for the ground users by optimizing the 3D UAVs’ coordinates and user association. To the best of our knowledge, the problem of total throughput maximization while optimizing UAV placement and user association in the HAP-UAV networks integration has not been examined yet in the literature. This novelty is highlighted in Table 1, which compares the features of our work proposed in this paper with existing references reviewed in Section 1.1.

Our main contributions can be described as follows:

- We formulate an optimization problem that aims to optimize user association and 3D UAV placement while maximizing the total system’s throughput.
- In order to improve the total throughput, we propose a clustering scheme based on the K-means clustering algorithm. Then, we investigate the optimal UAVs’ 3D position by exploiting an exhaustive search algorithm. The users–UAVs associations are local, which means that each UAV is associated with users from the same cluster only based on the best SNR.
- We develop a model in which the UAVs can serve users globally to reach the maximum total throughput, that is, UAVs can be associated with users outside their clusters’ boundary. Then, we apply the genetic algorithm [28] to obtain the optimized UAVs’ 3D coordinates and users’ association that maximizes the system’s total throughput.
- Finally, simulation results show that our designed genetic-algorithm-based allocation improves the performance and total throughput of the system by 32.5%, considering a coverage radius of 100 m compared to the total throughput when associating all users with the HAP.

### 1.3. Organization

This paper is organized as follows. In Section 2, we present the system model and problem formulation, including the communication model. Section 3 describes the proposed solution and Section 4 analyzes the performance of the proposed solution via simulation results. Finally, the paper is concluded in Section 5.

## 2. System Model and Problem Formulation

### 2.1. Communication Model

**Figure 1:**
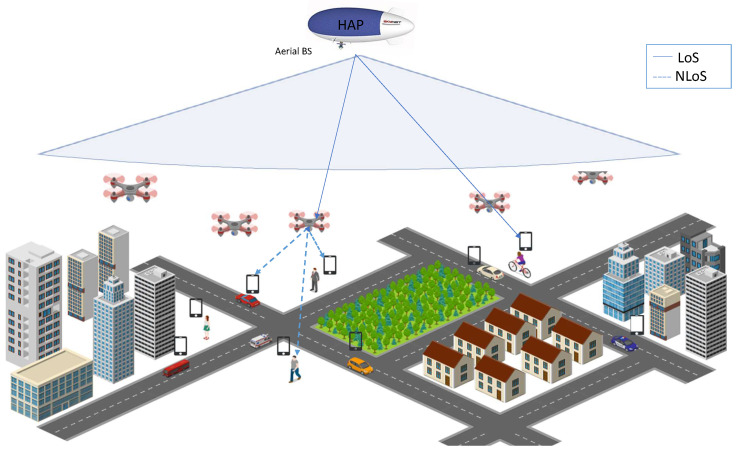
System model.

Our system model includes a single HAP *b* that acts as a BS and is assisted by a set M of UAVs acting as relays, i.e., M=1,2,...M. The system model contains many user equipments (UEs) in a dense area with many high-rise buildings, as shown in Figure 1. We also consider that both UAVs and UEs are equipped with a single orthogonal antenna. In contrast, the HAP is equipped with multiple orthogonal antennas to avoid interference. In addition to the above assumptions, we consider that all links between the HAP and UAVs, HAP and UEs, and UAVs and UEs are orthogonal channels. We further assume that the UAVs are either powered by the HAP [19] or by renewable energy or hybrid sources [29,30].

The system model consists of three channels:

- The LoS channel between the HAP and the UAVs ①.
- The NLoS channel between the UAVs and the UEs ②.
- The LoS channel between the HAP and the UEs ③.

The data transmission can take place in two ways, directly from the HAP to UEs via the LoS link ③ or from the HAP to UAVs to UEs through the LoS and NLoS links ① and ②. Note that the LoS channels are assumed due to the high altitude of HAP and the corresponding high elevation angle, while the NLoS channel is assumed due to the presence of many high-rise buildings and lower altitudes of UAVs. Let the locations of the HAP *b* UAV *m* and UE *u* be represented by $q_{b}$, $q_{m}$ and $q_{u}$, respectively, as follows:

$$\begin{array}{c} cq_{b}=\left(x_{b},y_{b},h_{b}\right),\\ q_{m}=\left(x_{m},y_{m},h_{m}\right),\\ q_{u}=\left(x_{u},y_{u},h_{u}\right), \end{array}$$

where $x_{b},y_{b}$ represent the horizontal location of the HAP *b*; $h_{b}$ is the height of the HAP *b*; $x_{m},y_{m}$ denote the horizontal location of UAV *m*; and $h_{m}$ is the height of UAV *m*. Similarly, $x_{u},y_{u}$ represent the horizontal location of UEs *u*, and $h_{u}$ is the height of UEs. The locations of UEs are fixed during the time and assumed to be distributed in the given area with uniform random heights within the range of 1.5–3.5 m. We assume the HAP hovers at a high altitude of 1.5 km; therefore, we consider the links ① and ③ to be LoS links, and thus their large-scale fading path loss follows the free space path loss. The overall channel gain for the link ③, between the HAP *b* and user *u*, can be modeled by

(1) $$G_{b,u}^{LoS}=G_{R}\cdot G_{T}{\left(\frac{c}{4\pi d_{b,u}f_{c}}\right)}^{2}{\left|h_{g,u}^{LoS}\right|}^{2},$$

while the overall channel gain for the link ① between the HAP *b* and the UAV *m* can be given as follows:

(2) $$G_{b,m}^{LoS}=G_{R}\cdot G_{T}{\left(\frac{c}{4\pi d_{b,m}f_{c}}\right)}^{2}{\left|h_{g,u}^{LoS}\right|}^{2},$$

where $G_{T}$ is the transmitter antenna gain, $G_{R}$ is the receiver antenna gain, *c* is the speed of light, and $f_{c}$ is the carrier frequency that is assumed to be 2 GHz for all links in this study. The factors ${\left(\frac{c}{4\pi d_{b,m}f_{c}}\right)}^{2}$ and ${\left(\frac{c}{4\pi d_{b,u}f_{c}}\right)}^{2}$ denote the propagation and path-loss for the LoS channel between the HAP *b* and UAV *m* and between the HAP *b* and user *u*, respectively. The factor $h_{g,u}^{LoS}$ represents the small-scale fading factor of the LoS links, corresponding to Rician fading. We assume that the Rician factor is the same and fixed for all channels. Let $d_{b,u}$ and $d_{b,m}$ denote the 3D distance between the HAP *b* at altitude $h_{b}$ and the user *u* at altitude $h_{u}$, and between the HAP *b* at altitude $h_{b}$ and the UAV *m* at altitude $h_{m}$ respectively. They are expressed as: $d_{b,u}=\sqrt{r_{b,u}^{2}+{\left(h_{b}-h_{u}\right)}^{2}},\;d_{b,m}=\sqrt{r_{b,m}^{2}+{\left(h_{b}-h_{m}\right)}^{2}}$, where $r_{b,u}$ is the horizontal distance between the HAP *b* and the user *u* and can be expressed as: $r_{b,u}=\sqrt{{\left(x_{b}-x_{u}\right)}^{2}+{\left(y_{b}-y_{u}\right)}^{2}}$, and $r_{b,m}$ denotes the horizontal distance between the HAP *b* and the UAV *m* and can be given as: $r_{b,m}=\sqrt{{\left(x_{b}-x_{m}\right)}^{2}+{\left(y_{b}-y_{m}\right)}^{2}}$.

This work does not consider the attenuation gain caused by the environmental effects such as rain, wind cloud, etc., since frequencies under 10 GHz are not affected significantly by the environmental effects [31]. Furthermore, we do not consider interference since the system includes a single HAP and the transmission is over orthogonal channels; as a result, the SNR for user *u* from HAP BS *b* can be expressed as follows:

(3) $$\begin{array}{c} \gamma_{b,u}=\frac{P_{T}G_{b,u}^{LoS}}{a_{u}BN_{0}}, \end{array}$$

where $P_{T}$ is the transmission power, and $a_{u}$ is the ratio of bandwidth allocation of user *u*. The sum of all users’ bandwidth allocation is equal to one to guarantee efficient use of the available bandwidth, $\sum_{u\in U}a_{u}=1$, where U=1,2,...U is the set of all the users, *B* is the available bandwidth, and $N_{0}$ is the Gaussian noise power spectrum density. The transmission data rate can be given as follows:

(4) $$C_{b,u}=a_{u}B\log_{2}\left(1+\gamma_{b,u}\right).$$

By substituting the values of $\gamma_{b,u}$ from Equation (3) and $G_{b,u}^{LoS}$ from Equation (1) in Equation (4), we obtain the LoS throughput, as illustrated below:

(5) $$C_{b,u}=a_{u}B\log_{2}\left(1+\frac{P_{T}G_{R}\cdot G_{T}{\left(\frac{c}{4\pi d_{b,u}f_{c}}\right)}^{2}{\left|h_{g,u}^{LoS}\right|}^{2}}{a_{u}BN_{0}}\right)$$

We consider a channel model in which link ② between each user *u* and each UAV *m* includes only the NLoS component; therefore the overall channel gain between UAV *m* and user *u* can be modeled by

(6) $$\begin{array}{c} G_{m,u}^{NLoS}=\frac{\beta_{0}\left(f_{c}\right){\left|h_{g,u}^{NLoS}\right|}^{2}}{{\left(d_{m,u}\right)}^{\alpha }}, \end{array}$$

where $\beta_{0}\left(f_{c}\right)$ is the path loss at the reference distance of 1 m, $h_{g,u}^{NLoS}$ is the small-scale fading factor of the NLoS link ② corresponding to Rayleigh fading, and α is the path loss exponent. Let $d_{m,u}$ denote the 3D distance between UAV *m* at altitude $h_{m}$ and user *u* at altitude $h_{u}$, and can be modeled as follows: $d_{m,u}=\sqrt{r_{m,u}^{2}+{\left(h_{m}-h_{u}\right)}^{2}}$, where $r_{m,u}$ is the horizontal distance between the UAV *m* and the user *u* and can be expressed as: $r_{m,u}=\sqrt{{\left(x_{m}-x_{u}\right)}^{2}+{\left(y_{m}-y_{u}\right)}^{2}}$. As a result, the resulting data rate of user *u* served by HAP *b* via UAV *m* can be written as

(7) $$\begin{array}{c} C_{m,u}^{\prime }=a_{u}B\log_{2}\left(1+\frac{P_{T}G_{b,m}^{LoS}G_{m,u}^{NLoS}}{a_{u}BN_{0}}\right), \end{array}$$

where $\frac{P_{T}G_{b,m}^{LoS}G_{m,u}^{NLoS}}{a_{u}BN_{0}}$ denotes the SNR of NLoS links. After substituting the values of $G_{b,u}^{LoS}$ from Equation (1) and $G_{m,u}^{NLoS}$ from Equation (6) in Equation (7), we obtain the following expression:

(8) $$\begin{array}{c} C_{m,u}^{\prime }=a_{u}B\log_{2}\left(1+\frac{\beta_{0}\left(f_{c}\right)P_{T}G_{R}.G_{T}{\left(\frac{c}{4\pi d_{b,m}f_{c}}\right)}^{2}|h_{g,u}^{LoS}{|}^{2}{\left|h_{g,u}^{NLoS}\right|}^{2}}{a_{u}BN_{0}{\left(d_{m,u}\right)}^{\alpha }}\right) \end{array}$$

### 2.2. Problem Formulation

In this paper, we have the following objectives:

1. Maximizing the system’s total throughput in the HAP-UAVs integrated network to guarantee maximum data transmission.
2. Obtaining the best UAV–user association that maximizes the throughput.
3. Finding the best UAVs placement, including the horizontal coordinates and their altitudes, to optimize the throughput.

First, the bandwidth allocation ratios are found through empirical studies. Then, we formulate an optimization problem that optimizes the UAV’s positions, and the user association with the HAP and the UAVs, maximizing the total system’s throughput. Based on the throughput definitions, the optimization problem is formulated as

(9) $$\begin{array}{ccc} \max_{x_{m},y_{m},h_{m},A} & \left(\sum_{m\in M}\sum_{u\in U^{\prime }}\left(a_{m,u}C_{m,u}^{\prime }\right)+\sum_{u\in (U-U^{\prime })}\left(\left(1-a_{m,u}\right)C_{b,u}\right)\right)\\ \mathrm{subject}\mathrm{to}C1: & N_{m}\leq N_{max},\; & \forall m\in M,\\ C2: & h_{min}\leq h_{m}\leq h_{max},\; & \forall m\in M,\\ C3: & x_{min}\leq x_{m}\leq x_{max},\; & \forall m\in M,\\ C4: & y_{min}\leq y_{m}\leq y_{max},\; & \forall m\in M,\\ C5: & a_{m,u}\in \left\{0,1\right\},\; & \forall m\in M,\forall u\in U, \end{array}$$

where $C_{b,u}$, given by Equation (5), and $C_{m,u}^{\prime }$, given by Equation (8), are the LoS and the NLoS individual throughputs of users, respectively. Let $U^{\prime }$ and ($U-U^{\prime }$) be the set of users associated with the UAVs and the HAP, respectively. The symbol *A* is the association matrix, where $A=\{a_{m,u}\},\forall m,\forall u$, and $a_{m,u}$ is a binary variable. If user *u* is served by UAV *m*, $a_{m,u}=1$; otherwise, $a_{m,u}=0$. Note that $a_{m,u}=0$ signifies that the user will be served by the HAP. In addition, $N_{max}$ is the UAV’s capacity, which is the maximum number of users that can be associated to it. Let $h_{min}$ and $h_{max}$ denote the UAV’s minimum and maximum height ∀m, respectively. Furthermore, $x_{min}/y_{min}$ and $x_{max}/y_{max}$ represent the minimum and maximum horizontal position bounds for all the UAVs, respectively. These bounds are based on the considered scene size. The indications of the constraints are as follows:

- Constraint C1 ensures that the number of users associated with UAV *m* is less than the maximum capacity.
- Constraint C2 indicates the height constraints of the UAV *m* to be in the range $\left[h_{min},h_{max}\right]$.
- Constraint C3 indicates that the *x* coordinate of the UAV *m* should in the range $\left[x_{min},x_{max}\right]$.
- Constraint C4 indicates that the *y* coordinate of the UAV *m* should in the range $\left[y_{min},y_{max}\right]$.
- Constraint C5 indicates that $a_{m,u}$ is a binary variable.

## 3. Proposed Solution

In this section, we present solutions for HAP-UAV–user association and 3D UAV placement considering throughput maximization. We first present a method for UAV–user association based on the best SNR. A method based on random association is also presented for comparison. Subsequently, we present an exhaustive search method to optimize the 3D placement of UAVs, where a certain percentage of users are associated with the UAV and the rest are associated with the HAP. This is followed by a joint 3D UAV placement and HAP-UAV–user association method based on the genetic algorithm. An analysis of the computational complexity and expected performance of the proposed methods is presented at the end of the section.

### 3.1. UAV–User Association

A collaboration framework between multiple UAVs and the HAP is considered in this section. Subsequently, the effect of the UAVs’ integration on the total throughput is examined. In addition, the best deployment scenario where the UAVs perform efficiently to improve the system’s throughput in cooperation with the HAP is discovered. It is assumed that the users are distributed uniformly in multiple clusters, whose total number is |M|, and one UAV is placed in each of them. Note that we consider the total number of cluster as equal to the total number of UAVs. A best-SNR-based algorithm is proposed, where $\frac{\left|U\right|}{\left|M\right|}$ users are associated with each UAV.

#### 3.1.1. Best-SNR-Based

Here it is assumed that the users are distributed in |M| clusters, i.e., one cluster for each UAV. A best-SNR-based algorithm is proposed, where the $\frac{\left|U\right|}{\left|M\right|}$ users having the highest SNR with respect to a UAV are associated to it. In addition, for comparison, we propose a random-SNR-based algorithm where all users are associated with UAVs randomly. Subsequently, using these algorithms, we aim to examine the effects of the user distribution radius on the total throughput. Our objective in this part is to examine and discover the radius range where the UAVs affect the total throughput positively. The algorithm details of the best-SNR-based algorithm are shown in Algorithm 1 and explained as follows:

- **Step 1:** As described in line 1, a single UAV is placed in the center of each cluster, assuming that users are uniformly distributed in |M| number of clusters in a given area.
- **Step 2:** In line 2, we calculate all possible NLoS SNR between UAVs and users to create a NLoS-SNR matrix with a size |U|×|M|.
- **Step 3:** At line 3, an empty vector, NLoS-SNR-Best, with size |U|×1 is created. In subsequent lines 4–13, a loop is created that iterates from first UAV to ${\left|M\right|}^{th}$ UAV. If the number of users associated with a UAV is less than |U|/|M|, find the maximum data rate of NLoS-SNR using Equation (8) between the considered UAV and users, and delete the row corresponding to the chosen user from the matrix NLoS-SNR so as not to choose it again since each user is associated with one UAV. The condition of the while loop ensures that each UAV is associated with the same number of users, i.e., $\frac{\left|U\right|}{\left|M\right|}$. Then, add the maximum value of throughput to NLoS-SNR-Best vector. After exiting the for loop, each UAV will be associated with |U|/|M| users based on the best SNR, so that we can compare their behavior.
- **Step 4:** Finally, in line 14, we calculate the total throughput by summing all the elements in NLoS-SNR-Best.

**Algorithm 1** Best-SNR-Based Association.1:Place a UAV in the center of each cluster2:Calculate NLoS-SNR(U,M) using Equation (8)3:I←1, NLoS-SNR-Best = zeros(|U|, 1)4:**for** m=1 to |M| **do**5:    count ←06:    **while** count <|U|/|M| **do**7:        [val,idx]←max(NLoS-SNR(:,m))8:        NLoS-SNR-Best(I,1)←val9:        I←I+110:      Delete NLoS-SNR(idx,:)11:      count ← count +112:   **end while**13:**end for**14:total-throughput ← sum(NLoS-SNR-Best)

#### 3.1.2. Random-SNR-Based

For reference, we also propose a random-SNR-based algorithm, which is described in Algorithm 2. The algorithm can be explained as follows:

- **Step 1:** As described in line 1, a single UAV is placed in the center of each cluster, assuming that the users are uniformly distributed in a specific number of clusters in a given area.
- **Step 2:** In line 2, we calculate all possible NLoS SNR ratios between UAVs and users to create a matrix NLoS-SNR of size |U|×|M|.
- **Step 3:** Create a vector, users-rand, containing a random permutation of the integers 1−|U| to arrange the elements of the NLoS-SNR matrix in a random order. An empty vector, Rand-NLoS-SNR, with size |U|×1 is also created.
- **Step 4:** Using users-rand, randomly selected |U|/|M| users are associated with each UAV before deleting their corresponding rows from NLoS-SNR in order not to associate them again with another UAV, as explained in lines 3–7.

**Algorithm 2** Random-SNR-Based Algorithm.1:Place a UAV in the center of each cluster2:Calculate NLoS-SNR(U,M) using (8)3:Generate users-rand(|U|), Rand-NLoS-SNR = zeros(|U|,1)4:**for** m=1 to |M| **do**5:    Rand-NLoS-SNR = NLoS-SNR(users-rand(1:|U|/|M|))6:    Delete users-rand(1:|U|/|M|)7:**end for**8:total-throughput ← sum(Rand-NLoS-SNR)

### 3.2. HAP-UAV–User Association

#### 3.2.1. Exhaustive Search

This section aims to find the optimal UAVs’ placement that maximizes the total throughput. We extend the system model proposed in Section 3.1.1, where users are served only by UAVs. Our developed model involves UAVs and HAP combinations so that they cooperate to serve users in a way that provides users with a maximum data rate. In the proposed model, users are served directly either by the HAP or UAVs. We assume that users association percentage is fixed with respect to a UAV in each cluster. The association between users and UAVs is examined and compared in the following two scenarios:

1. Best-SNR-based association.
2. Random-SNR-based association.

In addition, we assume that the association is equal and local, meaning that each UAV is associated with the same number of users in its cluster only. Furthermore, Rician and Rayleigh factors are assumed to be fixed. Initially, we assume that users are distributed uniformly. We use the K-means algorithm to divide the users into M clusters and find the centroid of each cluster. We place a UAV at the center of each cluster. We then propose an exhaustive search algorithm to find the 3D UAVs’ coordinates to maximize the system’s total throughput. Algorithm 3 shows the proposed exhaustive search algorithm. The steps of the algorithm are explained as follows:

- **Step 1:** In line 1, the K-means algorithm is applied to find the clusters with their centroids.
- **Step 2:** In line 2, we place a UAV in each centroid. This position is the initial position of the UAV.
- **Step 3:** In lines 3 to 6, for the $m^{th}$ UAV, we search the 3D UAV coordinates, $x_{m},y_{m},h_{m}$, in a range around its initial position. The range is denoted by $rng_{e}$ for $x_{m}$ and $y_{m}$. For $h_{m}$, we start searching at an initial height $h_{min}$ up to a maximum height $h_{max}$. The search step-sizes are step-x, step-y and step-h for $x_{m},y_{m},h_{m}$, respectively.
- **Step 4:** Next, for each $x_{m},y_{m},h_{m}$, the users in each cluster *m* are associated to the $m^{th}$ UAV based on the best or random SNR association. The total number of associated users are given by a percentage association. Subsequently, the sum of the associated users’ individual throughputs is calculated based on Equation (8). The HAP serves the remaining users in each cluster *m*, and the sum of their individual throughputs is calculated using Equation (5). Finally, the total throughput of users in a cluster *m* is calculated, which is the sum of the total throughput of users associated with the HAP and the total throughput of users associated with a UAV in a cluster *m*, as illustrated in line 7. In lines 11 to 14, we find the maximum value of the calculated total local throughput for UAV *m*, and the 3D UAV coordinates corresponding to this maximum throughput are selected as the optimal coordinates of the $m^{th}$ UAV. In line 15, the optimal 3D coordinates for all UAVs are obtained.
- **Step 5:** Finally, the system’s total throughput is calculated by summing the maximum local throughputs for each cluster, as shown in line 15.

**Algorithm 3** Exhaustive Search Algorithm.1:Determine clusters and centroids2:Initial 3D UAVs placement($x_{m},y_{m},h_{m}$) ← centroids3:**for** m=1 to M **do**4:    **for** $x_{m}\leftarrow \left[x_{m}-rng_{e}\;x_{m}+rng_{e}\right],\;step-x$ **do**5:        **for** $y_{m}\leftarrow \left[y_{m}-rng_{e}\;y_{m}+rng_{e}\right],\;step-y$ **do**6:           **for** $h_{m}\leftarrow \left[h_{min}\;h_{max}\right],\;step-h$ **do**7:               Calculate the local total throughput in cluster *m* based on (5) and (8) for the updated 3D coordinates, $x_{m},y_{m},h_{m}$. Save this throughput.8:           **end for**9:        **end for**10:    **end for**11:    Find maximum of the local total throughput for cluster *m*12:    Define 3D coordinates $x_{m},y_{m},h_{m}$ corresponding to the maximum local total throughput in cluster *m*13:**end for**14:Optimal UAVs placement is found15:Total throughput ← sum (local total throughput)

#### 3.2.2. Genetic-Algorithm-Based Allocation

In this section, we propose the final solution for our optimization problem Equation (9). The problem is a mixed-integer non-linear optimization problem, because the objective function is non-linear, and the solution variables include both binary variables, i.e., $a_{m,u}\forall m\in M,u\in U$, and continuous variables $x_{m},y_{m}$ and $z_{m},\forall m\in M$. Furthermore, the problem is non-convex due to the binary constraints. The solution to the problem requires the following steps:

1. Exploring through all 3D coordinates within the search space to select a position for each UAV,
2. For each UAV’s selected position, associate each user with either a UAV or the HAP, which is given by an association matrix of size |U|×|M|,
3. Find cumulative throughput corresponding to each UAV 3D coordinates and the user coordinates according to the association matrix,
4. Generate a new association matrix and repeat Step 3. The maximum number of combinations that can be generated is equal to $2^{\left|U|\times |M\right|}$. Some of these combinations can be ignored as they will violate the constraint C1 given in Equation (9),
5. Steps 1–4 are repeated for all possible UAV positions in the search space. The set of UAV positions and association matrix that gives the maximum cumulative throughput is the final solution.

The computational complexity of this problem will grow with the size of the search space, the number of UAVs, and the number of users. Hence, it will become computationally intractable in practical scenarios, and problems of this type are considered as NP-hard [32].

To solve this problem, we use a metaheuristic algorithm. Metaheuristic algorithms [33], such as the tabu search, PSO, ant colony optimization, simulation annealing, genetic algorithm, etc., are effective at solving difficult optimization problems such as non-convex problems, and can generally provide a solution close to the optimal one. We choose the genetic algorithm to jointly optimize the user association and UAV placement to maximize the system’s total throughput. The genetic algorithm is the most common optimization method due to its flexibility and implementation simplicity. It is inspired by the theory of natural evolution, where the fittest individuals are selected to reproduce the offspring of the next generation. The offspring will improve and have a better chance of survival if their parents have better fitness than their peers in the same generation. This reproduction process iterates until the best individuals are found. The process is as follows:

- **Initial population:** The optimization process starts with a set of individuals called the population. The characteristics of each individual are determined by variables called genes which join together to form a chromosome representing a solution. Genetic algorithm uses the initial solution to find the optimal ones.
- **Fitness function:** Fitness function is an evaluation metric that determines how close a solution is to the optimal solution. The fitness function generates a score for each individual. The higher the value, the best the solution.
- **Selection:** In this phase, the best individuals are selected as parents for mating to generate new individuals. In other words, the best individuals whose fitness values are the best are added to a mating pool to produce the next generation of solutions. The selection process consider selecting the fittest individuals among the previous and current generation to guarantee the survival of only the best solutions.
- **Crossover:** Crossover is the most significant phase in the genetic algorithm. Every two individuals in the mating pool are mated to generate a new individual in the next generation.
- **Mutation:** If there is a defect in the genes, it is passed on to the next generation because the offsprings carry their parents’ characteristics. Here comes the need for the mutation phase, where new characteristics are added to the new generation to have a wide spectrum of solutions and prevent premature convergence.
- **Convergence:** The previous four phases keep iterating until the algorithm terminates when there is no change in the fitness value. This means that the algorithm produces offspring that do not differ from their parents, meaning the best solutions to the given problem are found. Furthermore, the algorithm stops when a specified time is reached, or the specified number of generations has evolved.

The advantages of the genetic algorithm can be summarized as follows:

1. Genetic algorithm can be modified easily to optimize different problems.
2. Genetic algorithm performs well for multi-model problems that return a set of solutions.
3. It can discover optimal global solutions and avoid trapping in local solutions through the mutation phase, which guarantees a large and wide range of solutions.
4. It can optimize problems with multi-objective functions.
5. Genetic algorithm is a good choice for large-scale optimization problems.

**Figure 2:**
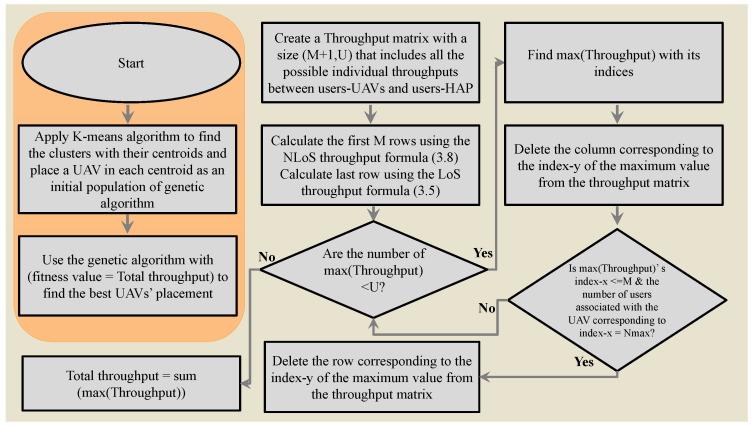
Genetic–algorithm–based allocation flowchart.

Algorithm 4 describes the proposed genetic algorithm and its fitness function, respectively. Figure 2 shows the corresponding flowchart. The steps of the genetic-algorithm-based allocation can be summarized as follows:

- **Step 1**: The K-means algorithm is applied to define |M| clusters with their centroids, as shown in line 1.
- **Step 2**: One UAV is placed initially in a centroid with 3D coordinates, $x_{m},y_{m},z_{m}$. Subsequently, the initial population of the genetic algorithm is set to the updated UAVs’ coordinates, as shown in line 2.
- **Step 3**: The genetic algorithm searches in a random space around the UAVs’ initial positions within a range $rng_{g}$. The lower bounds of $x_{m},y_{m}$, and $z_{m}$ are set to $x_{m}-rng_{g}$, $y_{m}-rng_{g}$, and $h_{min}$, respectively, as shown in line 3.
- **Step 4**: The upper bound of UAVs’ coordination is set to $x_{m}+rng_{g}$, $y_{m}+rng_{g}$, and $h_{max}$, respectively, as illustrated in line 4.
- **Step 5**: The genetic algorithm’s parameters are set to default.
- **Step 6**: The genetic algorithm is used to find the best placements of the UAVs. The genetic algorithm’s fitness function is defined as the total throughput found in the following steps.
- **Step 7**: An empty matrix, Throughput, with size equal to (|M|+1) ×|U| is created to include all the possible NLoS throughputs between all UAVs and users in the first |M| rows, and the LoS throughputs between the HAP and users in the last |M|+1 row, as shown in line 9.
- **Step 8**: The NLoS throughputs between all UAVs and users are calculated using Equation (8), as shown in lines 10 to 12.
- **Step 9**: The LoS throughput between all users and the HAP are calculated based on Equation (5), as shown in line 13.
- **Step 10**: An empty binary matrix A of size (|M|+1) ×|U| is created and initialized to zero. The first |M| rows indicate the association of $u^{th}$ user with the $m^{th}$ UAV if the $m^{th}$ row and $u^{th}$ column of A = 1. In a similar fashion, the last row indicates association of the user with the HAP by a value of 1. A Throughput-temp matrix that is equal to the Throughput matrix is created to search for the maximum users’ throughput, as shown in line 14.
- **Step 11**: In lines 15 to 22, we create a loop with a condition. If there is still a user that is neither associated with a UAV nor the HAP, the maximum value in the Throughput-temp matrix and its row and column indices are found. Subsequently, the corresponding indices in matrix A are set equal to 1. After that, the column corresponding to the column index of the maximum of Throughput-temp is deleted to ensure that the user is associated with only one UAV or the HAP. Next, if the user is associated with a UAV, which means that the row index of the maximum of Throughput-temp is less or equal to M, and if the UAV has reached its capacity, $N_{max}$, remove the row corresponding to the row index of the maximum of the Throughput-temp from the Throughput-temp matrix. The loop keeps on iterating until all users are served.
- **Step 12**: In line 23, the total throughput is calculated by summing the maximum throughput of users.

**Algorithm 4** Genetic-Algorithm-Based Allocation.1:Find |M| clusters and centroid2:Init-UAV-placement($x_{m},y_{m},z_{m}$) ← centroid3:LB ← [$x_{m}-rng_{g}\;y_{m}-rng_{g}\;h_{min}$]4:UB ← [$x_{m}+rng_{g}\;y_{m}+rng_{g}\;h_{max}$]5:Genetic algorithm’s parameters ← default6:Find UAVs’ placement using the genetic algorithm with the objective to maximize the total throughput, which is the output of the following function.7:
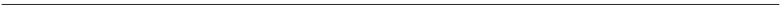
8:Function Throughput-calculation9:Throughput = zeros(|M|+1,|U|)10:**for** m=1to|M| **do**11:    Throughput(m,:) = NLoS throughput Formula (8), using positions given by one generation of the genetic algorithm.12:**end for**13:Throughput(|M| + 1, :) = LoS throughput Formula (5), using positions given by one generation of the genetic algorithm14:A = zeros(|M|+ 1, U), Throughput-temp = Throughput15:**while** sum(A)<|U| **do**16:    Find max(Throughput-temp) & its index-x and index-y17:    A(index-x,index-y) = 118:    Delete Throughput-temp(:,index-y)19:    **if** index-x ≤ |M|& sum(A(index-x,:)) = $N_{max}$ **then**20:        Delete Throughput-temp(index-x, :)21:    **end if**22:**end while**23:Total-throughput = sum(Throughput ⊙ A)24:return Total-throughput25:Endfunction

### 3.3. Computational Complexity of Exhaustive Search and Genetic-Algorithm-Based Allocation

Next, we compare the computational complexity of the exhaustive search and genetic-algorithm-based solutions. The main loop of the exhaustive search consists of iterative calculation of throughput for all the users corresponding to all the coordinates within the search range. Thus, one loop of this process involves calculation of a vector of size |U|, and is repeated for |M| UAVs. Therefore, the total number of calculations is $\left|M\right|\times \frac{2rng_{e}}{step-x}\times \frac{2rng_{e}}{step-y}\times \frac{h_{max}-h_{min}}{step-h}\times \left|U\right|$, where the second, third and fourth multiplicative terms represent the total number of points in the search grid in the *x*, *y* and *h* directions, respectively.

In the genetic-algorithm-based solution, a number of possible solutions, i.e., 3D coordinate values of the |M| UAVs are generated within the specified bounds iteratively, and the total throughput is calculated for each solution. This total throughput involves calculation of a matrix of size (|M|+1)×|U|. The total number of solutions is given by $N_{p}\times \left|M\right|$, where $N_{p}$ is the number of solutions generated per iteration by the genetic algorithm. This process is repeated for a fixed number of iterations, or generations as known in the terminology for the genetic algorithm, given by $N_{g}$. Therefore, the total number of calculations is equal to $(|M|+1)\times |U|\;\times \;(N_{p}\times \left|M|\right)\times N_{g}$.

In the next section, we carry out simulation experiments according to the parameters given in Section 4.1. With these parameters, the total number of search points in *x*, *y*, and *h* directions are approximately equal to 100 for the exhaustive search algorithm, M=5, and U=200. Consequently, the total number of calculations for the exhaustive search are approximately equal to $10^{9}$. For the genetic-algorithm-based solution, we use $N_{p}=200$ and $N_{g}=20$ based on trial and error. Hence, the total number of calculations in this case is equal to $2.4\times 10^{7}$, which is about 42 times less than that of the exhaustive search algorithm. Note that even if a value of $N_{g}=100$ is used, the total number of calculations is still less than 8 times that of the exhaustive search algorithm.

### 3.4. Analysis

In this section, we compare our proposed solutions for the HAP-UAV–user association and 3D placement. The exhaustive search solution is suboptimal since it only optimizes the 3D UAV placement though the exhaustive search solution and associates a fixed number of users with each UAV. However, the solutions obtained by the genetic-algorithm-based allocation are near-optimal because it jointly optimizes the UAV placement and user association.

In terms of fairness, the best-SNR-based association is used locally in the exhaustive search solution, which means that a user cannot be associated with a UAV outside its cluster even though there is a UAV that may provide better SNR to it in another cluster. On the other hand, the genetic-algorithm-based allocation solution provides more fairness as this search for the solution is global, which means that a user is associated with either a UAV or the HAP, depending on which entity may provide the best SNR to it. The associated UAV can be located in its cluster or outside as long as the UAV provides the best SNR to the user.

The exhaustive search solution consumes more time and is more complex since it has a loop for each coordinate to search over more positions. However, it provides better total throughput when the users are concentrated in small areas. On the other hand, the genetic-algorithm-based allocation solution can outperform the exhaustive search solution when users are spread in larger areas as it specifies the optimal user association to obtain the maximum throughput.

Finally, the exhaustive search solution associates all users according to the number of users specified to be associated with the UAV. On the other hand, the genetic-algorithm-based allocation solution associates the maximum number of users, which can be less than or equal to the capacity of UAVs.

We believe that the practical feasibility and implementation of the proposed solution would benefit from the Digital Twin concept [34] where based on models of the urban area under consideration as well as users’ historical density, the HAP-UAVs configurations can be investigated and suitable initial positions of the UAVs can be calculated. The Digital Twin can be implemented in the HAP. Subsequently, the UAVs positions can be adapted based on any change of users’ density.

## 4. Simulation and Results

### 4.1. Simulation Setup and Parameters

Simulations are carried out using MATLAB 9.12 R2022a. Table 2 lists the parameters used in our simulation. The initial UAV positions are denoted by $x_{m-init},\;y_{m-init}$, and $h_{m-init}$ in the table. User positions were generated such that |U|/|M| users are distributed uniformly around each UAV with a specified radius of 100 m creating five clusters. We consider that all channels have the same characteristics to be able to analyze other parameters. Therefore, we set the factors $h_{g,u}^{LoS}$ and $h_{g,u}^{NLoS}$ to 11.3267 and 1, respectively. Note that, in all figures, the x-axis denotes the distance between UAVs and users if the throughput is obtained through NLoS communication, or the distance between users and the HAP if the throughput is obtained through LoS communication.

The HAP is placed in the horizontal center at an altitude of 1.5 km to serve all users. Note that a HAP usually flies at an altitude that is about 10 times of that assumed in this paper. However, our system model is still applicable to the scenario with a very high altitude of the HAP, and the main difference in results in that scenario would be the association of a higher number of users to the UAVs, and a reduction in the overall cumulative throughput due to the larger HAP-UAV and HAP–user distance. It is found through experiments that the best bandwidth allocation of each user is approximately equal to $\frac{1}{\left|U\right|}=0.005$. Thus, each user is assigned a different bandwidth ratio equal to 0.005.

Subsequently, Algorithms 1 and 2 are simulated to study the effect of radius on the individual and total throughput in three cases: user–UAV best association, user–UAV random association, and user–HAP association. In Section 5 and Section 6, the UAVs are first placed in the initial positions given by the application of the K-means algorithm. Using Algorithm 3 that utilizes the exhaustive search in a 3D range around the initial positions of the UAVs, the optimal 3D UAVs’ coordinates are obtained in Section 5. Finally, in Section 6, Algorithm 4 is used in order to find the joint UAV placement and user association that maximizes the system’s total throughput.

### 4.2. UAV–User and HAP–User Association

In this part, we examine the effect of users’ distribution radius on the total and individual throughputs, as well as the behavior in the presence of UAVs and the HAP. We consider the following three scenarios:

1. All users are associated with the UAVs based on the best SNR.
2. All users are associated with the UAVs randomly.
3. All users are associated with the HAP.

In the first and second cases, we integrate five UAVs into our system to serve 200 users. Therefore, we place UAVs at their initial positions, $x_{m-init},\;y_{m-init}$, and $h_{m-init}$, as listed in Table 2. Subsequently, we distribute |U|/|M| users, i.e., 40 users, uniformly around each UAV in a certain radius to create five clusters. The users’ height ranges between 1.5 and 3.5 m. Thus, each UAV serves 40 users in its cluster. In the third case, it is assumed that the HAP serves all users and there is no UAV integration into the system. In all cases, Rician and Rayleigh factors are fixed to 11.3267 and 1, respectively. We carry out simulations to examine the total throughput obtained after association with each UAV with respect to different distribution radii.

After our simulations, we seek to answer the following questions:

- How does the radius affect the total throughput in the previous three cases?
- How does UAVs’ behavior differ with various radii in random and best association cases?
- How does the HAP behave with different radii?
- What radius is preferred in each case?

**Figure 3:**
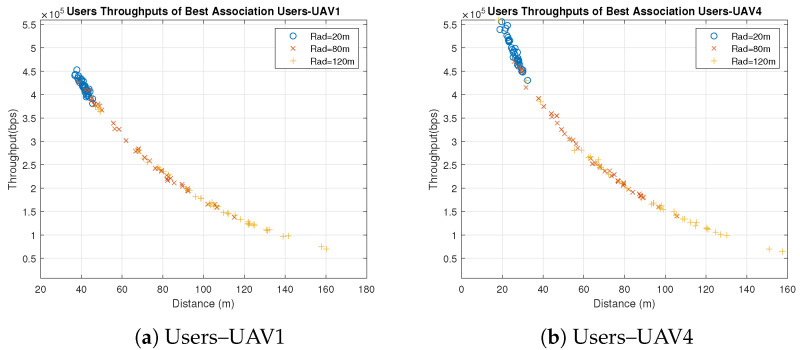
Individual Throughputs of Best SNR Association Users–UAV for Different Radii (Note: $h_{g,u}^{LoS}=11.3267,\;h_{g,u}^{NLoS}=1$).

**Figure 4:**
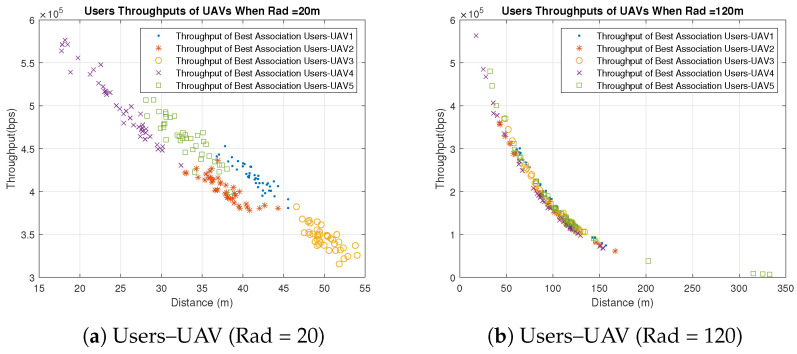
Individual Throughputs of Best SNR Association Users–UAV for Different Radii (Note: $h_{g,u}^{LoS}=11.3267,\;h_{g,u}^{NLoS}=1$).

Concerning the UAVs’ behavior, all UAVs behave similarly when users are associated with the UAVs based on the best SNR, because the only factor that affects the individual throughputs is the distance between the UAVs and users. All users are distributed uniformly in clusters with the same radius, and UAVs are placed in the clusters’ centers. Therefore, the significant factor that affects the distance is the UAVs’ heights which are different. Some UAVs have better throughput than others because of the differences in their heights and the slight differences in the users’ distribution, as shown in Figure 3 and Figure 4. Figure 3 shows the throughput of all users associated with UAV1 and UAV4 for distribution radii of 20 m, 80 m and 120 m. Figure 4 shows the total throughput of all users associated with each UAV for radii of 20 m and 120 m.

In Figure 3, we notice that the individual user throughputs from UAV4 are higher than those from UAV1 since the height of UAV4 is less than UAV1. On the other hand, it is observed in Figure 4a that the individual throughputs from UAV4 are between 4.5 × 10${}^{5}$ and 6 × 10${}^{5}$ bps, which are better than those from other UAVs. In addition, in Figure 4b, UAV4 demonstrates better individual throughputs than other UAVs. As a result, UAV4 performs the best with different radii compared to other UAVs because of its smallest height, which is 30 m. It is also observed that the individual throughput of each UAV and the radius are inversely proportional, which means that UAVs perform better with small radius.

**Figure 5:**
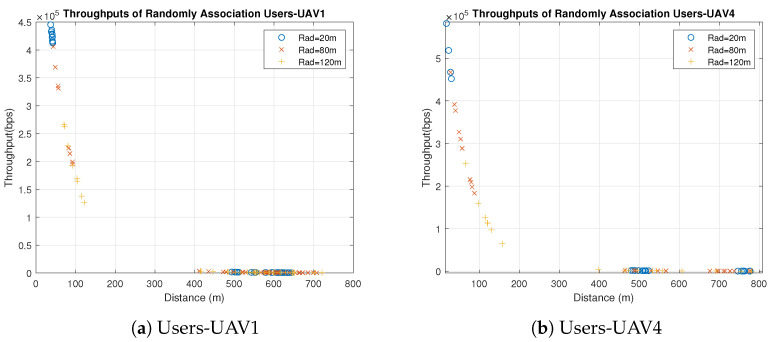
Individual Throughputs of Random Association Users-UAV for Different Radii (Note: $h_{g,u}^{LoS}=11.3267,\;h_{g,u}^{NLoS}=1$).

**Figure 6:**
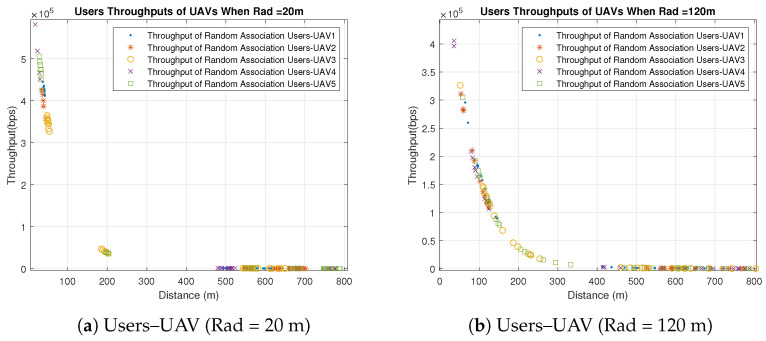
Individual Throughputs of Random Association Users–UAV for Different Radii (Note: $h_{g,u}^{LoS}=11.3267,\;h_{g,u}^{NLoS}=1$).

In the random users–UAV association, UAVs are randomly associated with users from different clusters, as illustrated in Figure 5 and Figure 6. It is noticed that most users have large distances comparing to the users associated with the UAVs based on the best SNR. The large distance impacts the individual throughput adversely, as shown in Figure 5. Most users have throughput close to zero due to the large distance. Therefore, the best SNR association outperforms the random association significantly.

Furthermore, in the random association, the UAVs’ heights do not have the same impact as in the best SNR association due to the random association with users from different clusters, which makes the horizontal distance to have more impact than the vertical one. Figure 6 shows that the UAVs’ behavior differs slightly even though they vary in their heights for the aforementioned reason. Similar to the best association, UAVs provide better individual throughputs with small radius, as illustrated in Figure 6. It is observed that both UAV1 and UAV4 provide better throughput when the radius is 20 m than compared to when the radius is 120 m.

**Figure 7:**
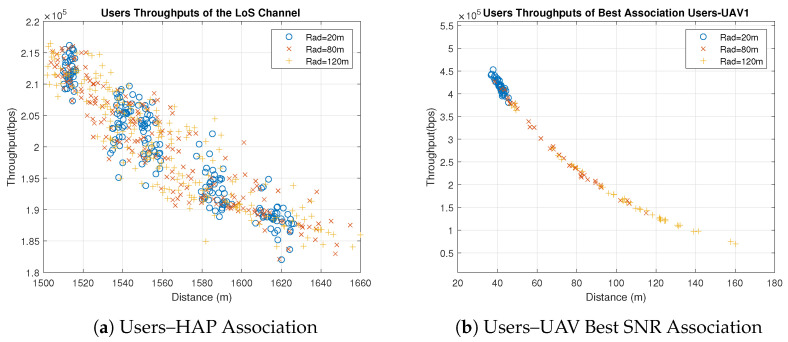
Individual Throughput Comparison between User–UAV Best SNR Association and User–HAP Association for Different Radii (Note: $h_{g,u}^{LoS}=11.3267,\;h_{g,u}^{NLoS}=1$).

**Figure 8:**
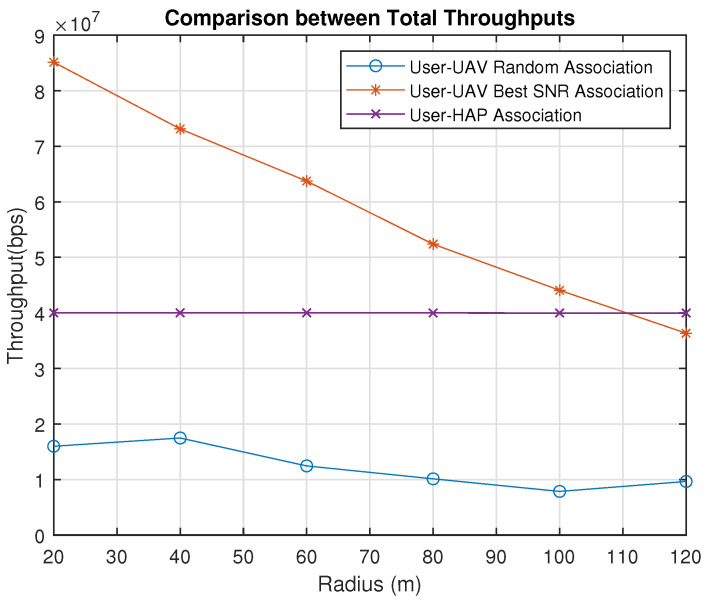
Total Throughput Comparison (Note: $h_{g,u}^{LoS}=11.3267,\;h_{g,u}^{NLoS}=1$).

Figure 7 shows the case where all users are associated with the HAP. It is observed that the users’ throughputs slightly vary with different radius compared to the best SNR association because the height of the HAP is considerably high compared to the radius. Therefore, the LoS total throughput stays almost constant, as illustrated in Figure 8. It is noticed that UAVs perform better than HAP when the radius is less than approximately 110 m. After the radius of 110 m, associating all UAVs with the HAP becomes the best choice to achieve better total throughput. On the other hand, the random association is the worst due to the random behavior, as illustrated in Figure 8.

## 5. Exhaustive Search

This section presents results with the Exhaustive Search approach where the UAV placement is optimized to maximize the total throughput while keeping the user percentage association fixed. Our optimized solution in this section is local, which means that after placing UAVs in the clusters’ centroid, UAVs are associated with users from the same cluster. The number of users associated is based on the user percentage association. In addition, all UAVs are associated with the same number of users in the clusters that may differ in size. Initially, users are distributed uniformly in five groups with a radius of 100 m. The UAVs–users association is examined in two cases, best-SNR-based and random association. We apply the proposed exhaustive search Algorithm 3, where we used the K-means algorithm to determine five clusters with their centroid. The initial position of the UAVs is the clusters’ centroid at the height of $h_{min}$ = 22 m.

**Figure 9:**
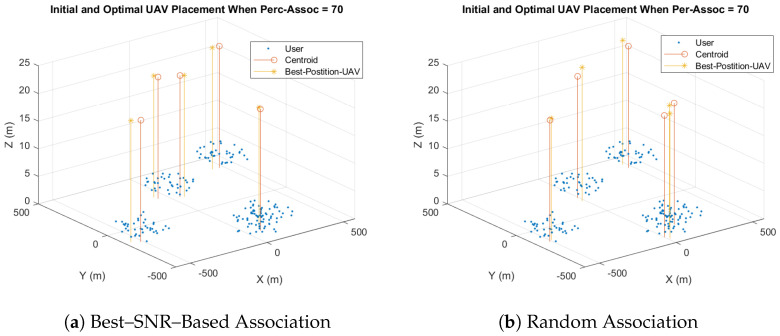
Initial and Optimal UAV Placement (Percentage Association (Perc–Assoc) = 70, Rad = 100 m).

We first set the user–percentage association to 70%, meaning that 70% of the users in each cluster are associated with the UAVs, and the remaining users are associated with the HAP. In addition, the range of the search space around each UAV, rng, is set to 50 m. Furthermore, the steps of the 3D search between each two searching points step−x, step−y, and step−h are 1 m, 1 m, and 0.28 m, respectively. Note that the random association is within one cluster, which means that each UAV serves users located in its cluster randomly, while in the best-SNR association, each UAV serves the users within the cluster that have the maximum SNR. The simulation results show that the optimal altitude of the UAVs is $h_{min}$, which is 22 m, in the best and random association. That is because the minimum altitude reduces the distance between users and UAVs, which in turn improves the throughput. Figure 9 shows UAVs’ initial and optimal 3D coordinates for the best-SNR and random-association for a radius of 100 m. In both cases, the optimal UAV placement is in the same cluster but with different 2D coordinates.

**Figure 10:**
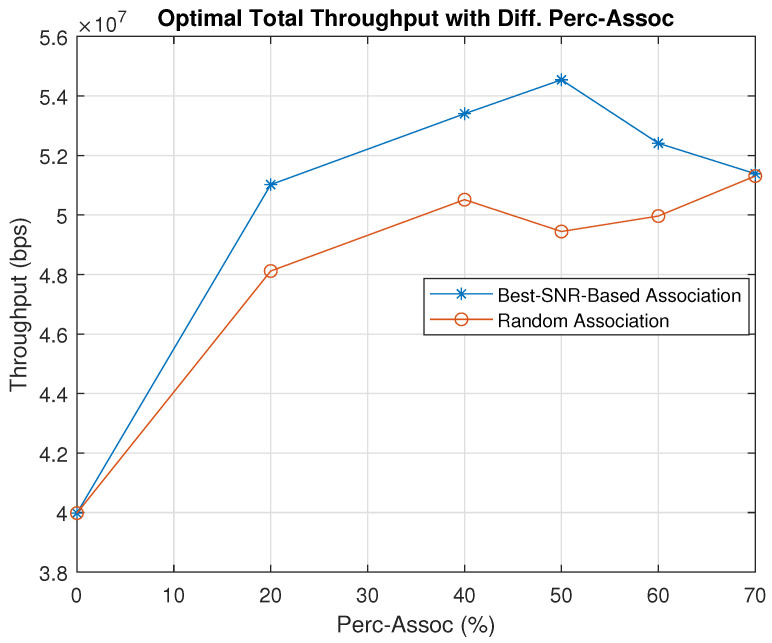
Total Throughput Comparison for Different Percentage Associations (Perc–Assocs), when Rad = 100 m.

To specify the best use cases of our exhaustive search algorithm, we apply our simulation with different user percentage associations and compare the optimal total throughput of the best and random association in Figure 10. The simulation results show that the total throughput of the best association considerably outperforms the random association for all percentage associations. In addition, we notice that the random association follows a random trend. However, the best association rises to reach the peak at 50% before falling steadily. We notice that the best user association that provides the maximum total throughput for a radius of 100 m is 50%. In other words, the best case for a radius of 100 m is to associate half of the users with the UAVs and the other half with the HAP. The results show that after integrating the UAVs into our network along with the HAP and applying our exhaustive search algorithm, the total throughput can increase up to 35% compared to the case where only a HAP is used.

## 6. Genetic-Algorithm-Based Association

**Figure 11:**
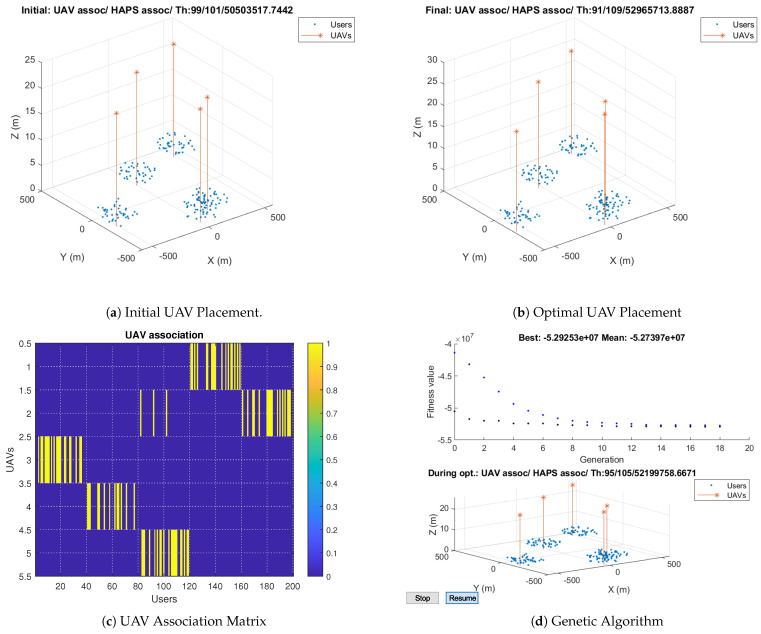
Genetic–Algorithm–Based Solutions when Rad = 100 m.

In this section, we present the results using the proposed Algorithm 4. The maximum UAV’s capacity, $N_{max}$, is set to 20. In addition, the search range, rng, and radius is set to 300 and 100, respectively. The simulation results are shown in Figure 11. Figure 11a illustrates the initial population of the genetic algorithm, where the user positions are the clusters’ centroids specified by the K-means algorithm. It also shows the initial total number of users associated with the UAVs and the HAP, as well as the total throughput obtained based on the initial positions. Figure 11b shows the optimal UAV placement obtained using the proposed Algorithm 4, user association, and total throughput. The optimal users–UAVs association is 91 users, less than 50% of the total users.

Figure 11c describes the UAV association matrix, which is a binary matrix. Each row in the matrix denotes the users associated with a UAV, represented by ones. The columns denote the users that are divided into five clusters. It is observed that UAV2 serves users from different clusters: a few users are served from cluster 3 in addition to the majority of the users served from cluster 5. This can help in providing a better overall throughput. The fitness value and the best individuals are illustrated in Figure 11d. It is observed that the genetic algorithm iterates until reaching convergence, where it finds the optimal solutions after 20 iterations. After applying our proposed genetic-algorithm-based allocation, the system’s throughput improvement for a radius of 100 m is around 32% compared to that obtained using only the HAP.

**Figure 12:**
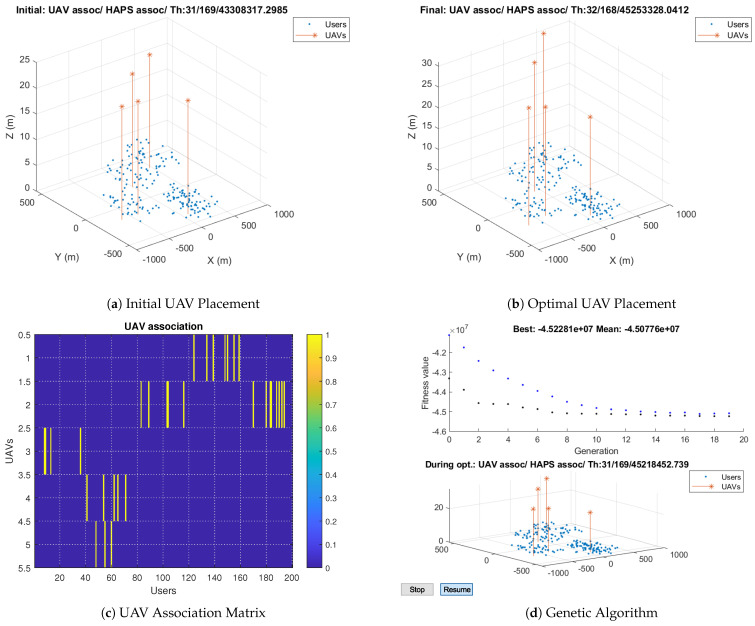
Genetic–Algorithm–Based Solutions when Rad = 200 m.

Next, we study our algorithm with a radius of 200 m. The simulation results show that 16% of the users are associated with the UAVs, while the remaining 85% are associated with the HAP, as shown in Figure 12b. In other words, the radius increase results in fewer users associated with UAVs in order to optimize the throughput. Figure 12c illustrates the users served by each UAV. A few users in each cluster are associated with a UAV, and UAVs serve users in two clusters. Figure 12a,d show the initial UAV placement and the optimal solution during optimization. The initial positions are the clusters’ centroid and genetic-algorithm-based allocation searches in the random space around the initial positions. It is noticed that the fitness value is improved during optimization until convergence to reach the best solution.

**Figure 13:**
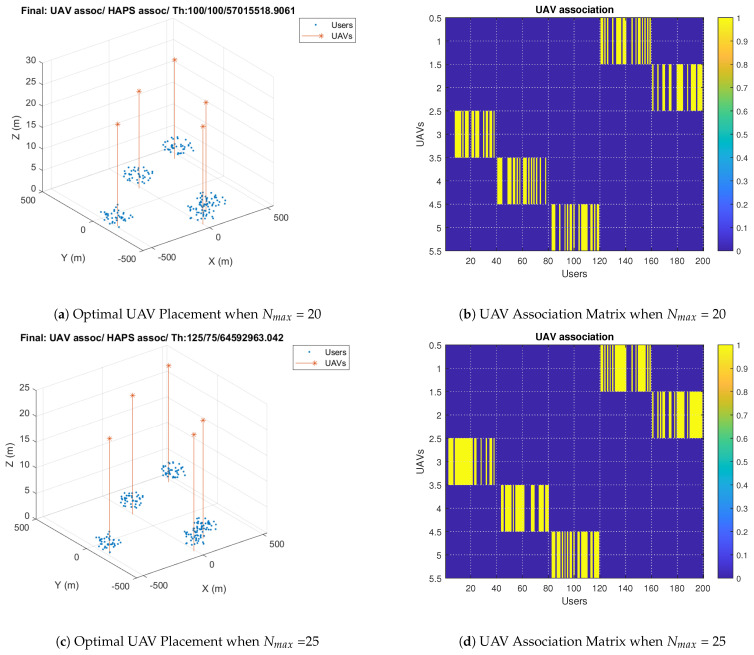
UAV Placement Comparison for Different Maximum Capacities when Rad = 60 m.

In Figure 13, we show results with a radius of 60 m and compare the results with two different maximum number of users that can be associated with each UAV. It can be noticed that for a smaller radius of 60 m, there is a need to associate more users with the UAVs to improve the total throughput, but the maximum UAVs’ capacity can prevent this from happening. It is noticed that with a capacity of 20 users, the number of users associated with the UAVs is 100, which becomes 125 users when increasing the capacity to 25, as illustrated in Figure 13a,c. When the maximum capacity of the UAV is 20, a UAV cannot be associated with more than 20 users, as illustrated in Figure 13a,b. On the other hand, increasing the maximum UAVs’ capacity allows the UAVs to serve more users and improve the total throughput, as shown in Figure 13c,d.

**Figure 14:**
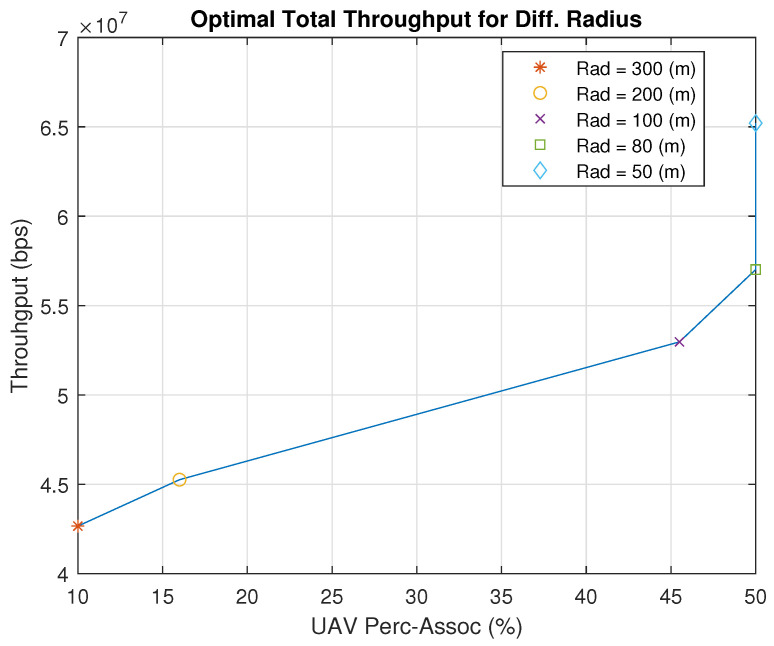
Total Throughput Comparison.

Figure 14 shows how our proposed genetic-algorithm-based solution performs with respect to varying user percentage association and different radii. The y-axis of the figure shows the maximum throughput received corresponding to a specified radius and user percentage association. Here, the user percentage association is with respect to the total number of users, e.g., 50% means that the maximum number of users that can be associated is 20 out of a total of 40 users for a UAV. With a radius of more than 80 m, the algorithm finds the optimal user percentage association to maximize the throughput. However, with a radius less than 80 m, it cannot associate more users with the UAVs to increase the throughput. Therefore, we found that for radii of 80 m and 50 m, the optimal percentage is 50%, which is the maximum possible percentage.

**Figure 15:**
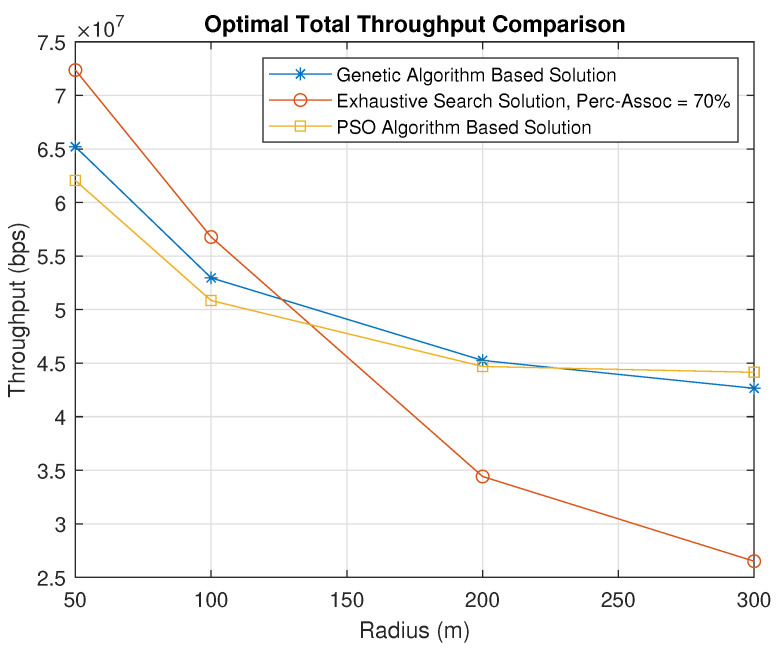
Total Throughput Comparison Between Genetic–Algorithm–Based, Exhaustive Search, and PSO–Based Solutions.

Figure 15 describes the best use cases of the exhaustive search algorithm and the genetic-algorithm-based solution. The UAV percentage association denotes the percentage of users associated with all UAVs. It is observed that when the users are concentrated in small areas with a radius of 100 m and below, the exhaustive search algorithm outperforms the genetic algorithm since the maximum number of users should be assigned to UAVs. The exhaustive search algorithm dedicates 70% of the users in each cluster to be served by a UAV and then finds the optimal UAV placement. On the other hand, when the radius exceeds 100 m, the genetic-algorithm-based solution performs better as it looks for the optimal user percentage association as well as the optimal UAV placement. Therefore, for a radius over 100 m, it is better to use the genetic-algorithm-based solution, while it is better to use the exhaustive search solution for a radius less than 100 m.

**Figure 16:**
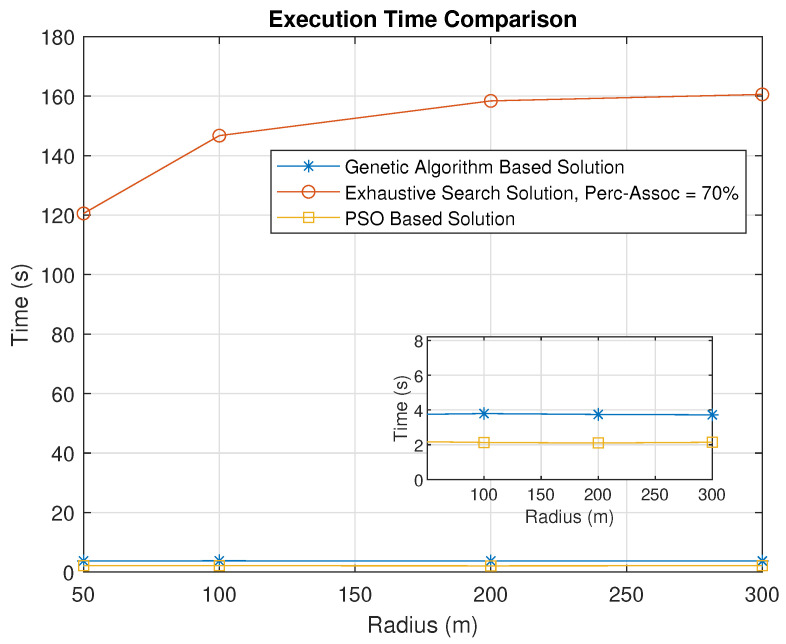
Execution Time Comparison Between Genetic–Algorithm–Based, Exhaustive Search, and PSO–Based Solutions.

We also compare the performance using a PSO-algorithm-based solution. The throughput obtained using the PSO algorithm is almost similar to that obtained using the genetic-algorithm-based solution, with some small variations. Thus, our proposed solution is not dependent on the user of a particular metaheuristic algorithm, and other metaheursitic algorithms can also be used. A comparison of the execution times for the exhaustive search, genetic algorithm and PSO-algorithm-based solutions is shown in Figure 16. As expected based on the analysis presented in Section 3.3, the exhaustive search takes significantly longer than the genetic-algorithm-based solution. Furthermore, the former algorithm’s execution time does not show any major variations with respect to the increasing radius, which indicates its suitability in practical scenarios. The PSO-algorithm-based solution is slightly faster than the genetic-algorithm-based solution.

## 7. Conclusions

This paper investigated HAP-UAV integrated networks in wireless communications. The objective of this study was to optimize 3D UAV positions and user associations with the goal of maximizing the cumulative throughput of the system. The optimization was solved using the K-means algorithm, and an exhaustive search approach as well as a genetic-algorithm-based approach. The simulation results demonstrated that the key factor affecting the benefit obtained using a UAV was the radius within which the users were distributed around the UAV. The benefit of the UAV increased with a decreasing radius. In other words, UAVs had more positive influence on total throughput when serving small areas with a large density of users. The proposed exhaustive search algorithm aimed to discover the optimal 3D UAV positions, while associating a specified number of users around each UAV. It provided better solutions than the proposed genetic-algorithm-based allocation with a small radius as it permitted more users to be associated with the UAVs. On the other hand, the genetic-algorithm-based allocation could achieve joint optimal 3D UAV placement and user association when serving users distributed in large areas since it identified the best user association to maximize the total throughput. Furthermore, the genetic-algorithm-based allocation had a lower computational complexity compared to the exhaustive search algorithm.

We have made some assumptions in this paper, which can be addressed in future work. These assumptions as well as our outlook are as follows: (1) We concentrate on the UAV placement and the user association problem, while assuming the absence of any interference between users. We also do not consider any energy constraints with respect to the UAV. An extension of this work can involve considering the interference as well as minimizing the energy consumption of the UAVs. (2) We consider all channels to have identical characteristics. A future area of research is to optimize total throughput considering different characteristics of each channel. Furthermore, the effect of the attenuation gain caused by the environmental effects can be investigated with respect to its influence on total throughput. (3) We assume the users to be static and the number of UAVs to be fixed in this work. A possible extension of this work will involve considering mobile users and subsequently optimizing the system’s total throughput using reinforcement learning. A new optimization problem that minimizes the maximum number of UAVs needed to maximize the throughput can also be formulated.

## Figures and Tables

**Table 1:** Comparison of existing references with the proposed work. The links considered by a reference are shown by “✓”.

Ref.	UAV-User	HAP-User	HAP-UAV	Objectives
[1]	✓		✓	Min. computing latency by optimizing the offloading decision, transmit power, and uplink signal splitting ratio
[5]	✓		✓	UAV placement to optimize channel and power allocation between UAVs and users
[7]	✓			UAV placement, min. no. of deployed UAVs, max. coverage area
[8]	✓			UAV placement, min. no. of deployed UAVs
[9]	✓			UAV placement, max. no. of users and sum data rate considering the backhaul peak rate of UAVs
[10]	✓			UAV placement, enhance the UAV-D2D network using NOMA
[11]	✓			UAV placement and bandwidth allocation to min. cumulative power
[12]	✓			UAV placement, max. no. of covered users and min. the transmission power
[13]	✓			Max. uplink & downlink sum rate using full duplex communication in UAVs
[14]	✓			UAV-user association to min. transmit power of users
[15]	✓			Investigate the effect of altitude and no. of UAVs on the cumulative rate maximization
[16]	✓			Max. overall system throughput
[17]	✓			UAV placement and user association to max. throughput considering LoS and NLoS links between UAVs and users
[18]	✓			UAV placement, user association, and bandwidth allocation considering wireless backhaul link between UAVs & a macro BS
[19]	✓		✓	Max. overall utility of UAVs by offloading tasks to a terrestrial computing node, HAP is used to charge UAVs via laser beams
[20]		✓		Optimize resource allocation by offloading decision, signal splitting rate, and transmit power in a HAP-vehicular computing network
[21]	✓	✓		Optimizing device association, partial offloading, and communication resources for IoT-HAP-UAV network
[22]	✓	✓		Power management to min. distortion in multimedia streaming, both UAVs and HAP are assisted by a ground BS
[23]		✓		Placing RIS on HAP, max. no. of users, min. power consumption
[24]	✓	✓		Min. processing time by optimizing the computing task offloading factor from ground vehicles to UAVs or HAP
[26]	✓		✓	Channel allocation to max. sum data rate
[27]	✓	✓		UAV placement, load balancing among UAVs, fairness in data rate provisioning
This work	✓	✓	✓	UAV placement, max. total system throughput

**Table 2:** Simulation parameters.

Notation	Value
M	|M|=5
U	|U|=200
$\left(x_{b},y_{b}\right)$	$x_{b}=0,y_{b}=0$
$h_{b}$	1.5 km
$x_{m-init}$	[2, −500, −9, 478, 158] m
$y_{m-init}$	[214, −151, −414, 379, −318] m
$h_{m-init}$	[60, 52, 85, 30, 46] m
$h_{u}$	[1.5, 3.5] m
$rng_{e}$	50 m
$rng_{g}$	300 m
$G_{T}$	17 dBi
$G_{R}$	16 dBi
$P_{T}$	23 dBm
*c*	3 × $10^{8}$ m/s
$f_{c}$	2 GHz
$h_{g,u}^{LoS}$	11.3267
$h_{g,u}^{NLoS}$	1
$N_{0}$	−134 dBm/Hz
*B*	10 GB
$\beta_{0}$	4
α	3.7
$h_{min}$	22 m
$h_{max}$	50 m
$N_{max}$	20

## Data Availability

Data can be simulated according to parameters mentioned in Section 4. Data sharing is not applicable to this article.

## Abbreviations

The following abbreviations are used in this manuscript:

UAV	Unmanned aerial vehicles
BS	Base station
LoS	Line-of-sight
NLoS	Non-line-of-sight
HAP	High altitude platform
LEO	Low earth orbit
A2G	Air-to-ground
QoS	Quality of service
SNR	Signal-to-noise ratio
PSO	Particle swarm optimization
D2D	Device-to-device
NOMA	Non-orthogonal multiple access
BLLL	Binary log-linear learning algorithm
RIS	Reflective intelligent surfaces
UE	User equipment

## List of symbols

M	Set of UAVs
U	Set of users
$d_{b,u}$	3D distance between the HAP *b* and user *u*
$d_{b,m}$	3D distance between the HAP *b* and UAV *m*
$d_{m,u}$	3D distance between UAV *m* and user *u*
$\left(x_{b},y_{b}\right)$	Horizontal location of the HAP *b*
$\left(x_{m},y_{m}\right)$	Horizontal location of the UAV *m*
$x_{m-init}$	Initial values of $x_{m}$ of the UAVs
$y_{m-init}$	Initial values of $y_{m}$ of the UAVs
$\left(x_{u},y_{u}\right)$	Horizontal location of the user *u*
$h_{b}$	Height of HAP *b*
$h_{m}$	Height of UAV *m*
$h_{u}$	Height of a user *u*
$r_{b,u}$	Horizontal distance between the HAP *b* and a user *u*
$r_{b,m}$	Horizontal distance between the HAP *b* and UAV *m*
$r_{m,u}$	Horizontal distance between UAV *m* and user *u*
$G_{b,u}^{LoS}$	LoS-channel gain between the HAP *b* and user *u*
$G_{b,m}^{LoS}$	LoS-channel gain between the HAP *b* and UAV *m*
$G_{m,u}^{NLoS}$	NLoS-channel gain between UAV *m* and user *u*
$G_{T}$	Transmitter antenna gain
$G_{R}$	Receiver antenna gain
$P_{T}$	Transmit power of the HAP
*c*	Speed of light
$f_{c}$	Carrier frequency
$h_{g,u}^{LoS}$	Small-scale fading factor of the LoS link between user *u* and the HAP
$h_{g,u}^{NLoS}$	Small-scale fading factor of the NLoS link between user *u* and UAV *m*
$\gamma_{b,u}$	Signal to noise ratio(SNR) at the location of user *u* from HAP *b*
$N_{0}$	Gaussian noise power spectrum density
$a_{u}$	Bandwidth ratio allocation of user *u*
*B*	Available bandwidth ∀u
$C_{b,u}$	Achievable data rate at user *u* served by the HAP *b*
$C_{m,u}^{\prime }$	Achievable data rate at user *u* served by the HAP through UAV *m*
$\beta_{0}$	NLoS link reference path loss
α	NLoS link path loss factor
$N_{m}$	Number of users associated with UAV *m*
$N_{max}$	Maximum capacity of UAV ∀m
$h_{min}$	Minimum height of UAV ∀m
$h_{max}$	Maximum height of UAV ∀m
*A*	Users–UAVs association vector
$a_{m,u}$	A binary variable equal to 1 if user *u* is assigned to UAV *m*, otherwise 0
$q_{b}$	Location of the HAP *b*
$q_{m}$	Location of UAV *m*
$q_{u}$	Location of user *u*
